# Virocidal activity of Egyptian scorpion venoms against hepatitis C virus

**DOI:** 10.1186/s12985-015-0276-6

**Published:** 2015-03-24

**Authors:** Alaa MH El-Bitar, Moustafa MH Sarhan, Chie Aoki, Yusuke Takahara, Mari Komoto, Lin Deng, Mohsen A Moustafa, Hak Hotta

**Affiliations:** Department of Zoology, Faculty of Science, Al-Azhar University, Assiut, Egypt; Division of Microbiology, Kobe University Graduate School of Medicine, 7-5-1 Kusunoki-cho, Chuo-ku, Kobe, 650-0017, Hyogo Japan

**Keywords:** Hepatitis C virus, HCV, Antiviral activity, Scorpion venom, *Scorpio maurus palmatus*, Egypt

## Abstract

**Background:**

Hepatitis C virus (HCV) is a major global health problem, causing chronic hepatitis, liver cirrhosis and hepatocellular carcinoma. Development of well-tolerated regimens with high cure rates and fewer side effects is still much needed. Recently, natural antimicrobial peptides (AMPs) are attracting more attention as biological compounds and can be a good template to develop therapeutic agents, including antiviral agents against a variety of viruses. Various AMPs have been characterized from the venom of different venomous animals including scorpions.

**Methods:**

The possible antiviral activities of crude venoms obtained from five Egyptian scorpion species (*Leiurus quinquestriatus, Androctonus amoreuxi, A. australis, A. bicolor* and *Scorpio maurus palmatus)* were evaluated by a cell culture method using Huh7.5 cells and the J6/JFH1-P47 strain of HCV. Time-of-addition experiments and inactivation of enzymatic activities of the venoms were carried out to determine the characteristics of the anti-HCV activities.

**Results:**

*S. maurus palmatus* and *A. australis* venoms showed anti-HCV activities, with 50% inhibitory concentrations (IC_50_) being 6.3 ± 1.6 and 88.3 ± 5.8 μg/ml, respectively. *S. maurus palmatus* venom (30 μg/ml) impaired HCV infectivity in culture medium, but not inside the cells, through virocidal effect. The anti-HCV activity of this venom was not inhibited by a metalloprotease inhibitor or heating at 60°C. The antiviral activity was directed preferentially against HCV.

**Conclusions:**

*S. maurus palmatus* venom is considered as a good natural source for characterization and development of novel anti-HCV agents targeting the entry step. To our knowledge, this is the first report describing antiviral activities of Egyptian scorpion venoms against HCV, and may open a new approach towards discovering antiviral compounds derived from scorpion venoms.

## Introduction

Hepatitis C virus (HCV) infection is a major global health problem, with estimated more than 170 million infected individuals worldwide. HCV is an enveloped, positive-strand RNA virus that belongs to the *Hepacivirus* genus of the *Flaviviridae* family [1-3]. HCV infection is the serious cause of chronic hepatitis, hepatic steatosis, liver cirrhosis and eventually hepatocellular carcinoma after a few decades. There is no anti-HCV vaccine available and therapeutic options are still limited. The current standard therapy, which is based on pegylated interferon and ribavirin, is only partially effective, resulting in a sustained virological response (SVR) in about 50% of patients and has considerable side effects [1,3]. Recently, HCV NS3 protease inhibitors and NS5A inhibitors have been approved for clinical use and SVR rates have improved to reach 70% or higher [4-8]. However, these therapies are quite expensive and will probably not be accessible for all patients worldwide. For this reason, the development of new classes of safe and inexpensive antiviral compounds with improved efficacy is still needed for treatment of HCV infections.

Recently, natural antimicrobial peptides (AMPs) are attracting more attention as therapeutic agents against a variety of microbes including antibiotics-resistant strains [9,10]. Most AMPs share certain common features such as being small peptides of 10 to 50 amino acid residues, containing positive charge of 2 to 9 residues and an amphipathic structure [11-13]. These peptides exhibit a broad spectrum of antiviral and antibacterial activities, with direct or indirect microbicidal activities [12]. AMPs have been isolated from venomous animals including scorpions. Scorpion venoms consist of a cocktail of biologically active peptides that represent a tremendous potential for use in drug design and development [14-17]. Most scorpion venom peptides are composed of 20 to 75 amino acid residues while certain proteins, enzymes, consist of 120 to 370 residues [17]. Scorpion venom peptides show a vast array of biochemical activities and pharmacological functions. They can be classified into two classes, i.e., disulfide-bridged and non-disulfide-bridged peptides [18-20]. AMPs found in scorpion venoms are paid more and more attention due to their unique biological activities that can potentially be used as broad-spectrum antiviral agents [9,21-28]. Scorpion venom AMPs are positively charged amphipathic peptides and can be divided into three structural categories: (i) cysteine containing peptides with disulfide bridges; (ii) peptides with an amphipathic α-helix but lacking cysteine residues and (iii) peptides rich in certain amino acids such as proline and glycine [29].

The list of Egyptian scorpions currently includes 24 species classified under 13 genera within four different families, Buthidae, Diplocentridae, Euscorpiidae and Scorpionidae [30]. In the present study, we screened crude venoms obtained from five Egyptian scorpion species, *Leiurus quinquestriatus, Androctonus amoreuxi, A. australis, A. bicolor* and *Scorpio maurus palmatus,* for possible anti-HCV activities using an HCV cell culture system. We report here that crude venoms of *S. maurus palmatus*, and *A. australis* to a lesser extent, possess antiviral activities against HCV. To our knowledge, this is the first report describing anti-HCV activities of Egyptian scorpion venoms.

## Results

### Screening of anti-HCV activities of scorpion venoms

**Figure 1:**
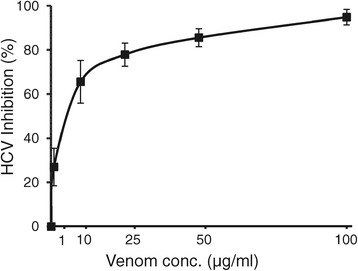
**Dose-dependent anti-HCV activity of** ***S. maurus palmatus*** **venom.** A fixed amount of HCV was mixed with serial dilutions of *S. maurus palmatus* venom and inoculated to Huh7.5 cells at a multiplicity of infection of 0.5 pfu/cell. After virus adsorption, the cells were cultured with the same concentrations of the venom for 46 hr. The culture supernatants were harvested and titrated for virus infectivity. Percent inhibitions of HCV infectivity by the venom at the concentrations of 0.1 to 100 μg/ml are shown. Data represent means ± SEM of the data obtained from two independent experiments.

Anti-HCV activities of crude venoms of five Egyptian scorpion species were tested. As shown in Table 1, *A. australis* and *S. maurus palmatus* showed anti-HCV activities, with IC_50_ being 88.3 ± 5.8 and 6.3 ± 1.6 μg/ml, respectively. Their CC_50_ were >300 and >100 μg/ml, respectively, with their selectivity indexes (SI; CC_50_/IC_50_) being >3.4 and >15.8, respectively. Crude venoms of the other three scorpion species did not exhibit significant anti-HCV activities at the concentration of 100 μg/ml. Dose-dependent anti-HCV activity of *S. maurus palmatus* is shown in Figure 1.

**Table 1:** **Antiviral activity (IC**_**50**_ **) against HCV, cytotoxicity (CC**_**50**_ **) and selectivity index (SI) of crude venoms of five Egyptian scorpion species tested in this study**

**Species**	**IC**_**50**_ **(μg/ml)** ^**a**^	**CC**_**50**_ **(μg/ml)** ^**a**^	**SI**
*Leiurus quinquestriatus*	>100	>100	na
*Androctonus amoreuxi*	>100	>100	na
*Androctonus australis*	88.3 ± 5.8	>300	>3.4
*Androctonus bicolor*	>100	>100	na
*Scorpio maurus palmatus*	6.3 ± 1.6	>100	>15.8

^**a**^: Data represent means ± SEM of the data obtained from two independent experiments using the J6/JFH1-P47 strain of HCV.

na: Not applicable.

### HCV inhibitory mechanisms of the *S. maurus palmatus* venom

**Figure 2:**
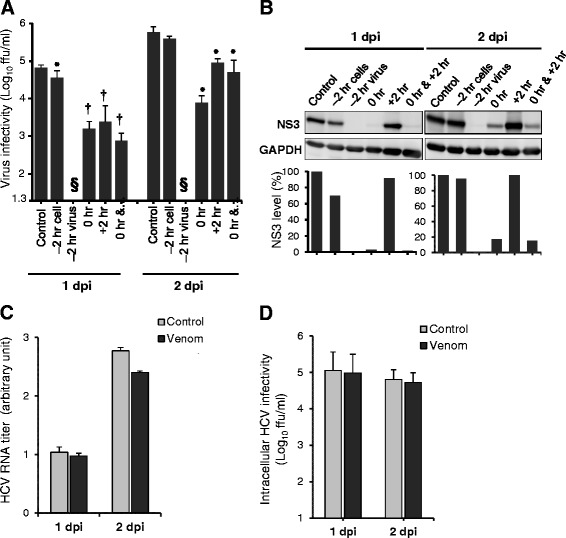
**Analysis of mode-of-action of** ***S. maurus palmatus*** **venom** ***.*** Huh7.5 cells were infected with HCV and treated with *S. maurus palmatus* venom (30 μg/ml) at different time points as indicated **(A, B)** or left untreated as a control. **(A)** Amounts of HCV infectious particles in the supernatants. Data represent means ± SEM of the data obtained from two independent experiments. *, *P* <0.05; †, *P* <0.001, compared with the untreated control; §, below the detection limit; dpi, days post-infection. **(B)** HCV NS3 protein accumulation in the cells. Virus-infected cells were subjected to immunoblot analysis using monoclonal antibody against the HCV NS3 protein at 1 and 2 days post-infection. GAPDH served as an internal control to verify equal amounts of sample loading. Signal intensities of NS3 were normalized to the corresponding GAPDH signal. **(C)** Amounts of HCV RNA in the cells. The venom treatment was done only during the post-inoculation (+2 hr) step. HCV RNA amounts were normalized to GAPDH mRNA expression. **(D)** Amounts of HCV infectious particles inside the cells. The venom treatment was done only during the post-inoculation (+2 hr) step. Virus-infected cells were subjected to 3 cycles of freezing and thawing at −80°C and 37°C, respectively, and HCV infectivity inside the cells was measured.

To further explore the mode of action of the *S. maurus palmatus* venom, time-of-addition experiments were performed. In brief, *S. maurus palmatus* crude venom (30 μg/ml) was added to the virus and/or cells at different time points relative to virus inoculation, as described in the Methods section: (i) pre-treatment of cells for 2 hr (−2 hr cells). This experiment examines whether there is any interaction between the venom and the cells. (ii) pre-treatment of virus for 2 hr (−2 hr virus). This experiment examines the possible virocidal activity of the venom. (iii) co-treatment of cells and virus during virus inoculation for 2 hr (0 hr). This experiment examines the antiviral effect at the entry step. (iv) treatment of virus-infected cells during post-inoculation for 46 hr (+2 hr). This experiment examines the antiviral effect during the post-entry step. (v) co-treatment and post-inoculation (0 hr & +2 hr) as a positive control. For each set of experiments, HCV infectivity in culture supernatants was determined and compared with each other. The result revealed that pre-treatment of virus for 2 hr (−2 hr virus) markedly suppressed production of HCV infectious particles in culture supernatants while pre-treatment of cells for 2 hr (−2 hr cells) only marginally suppressed it (Figure 2A). This suggested the possibility that *S. maurus palmatus* venom had direct virocidal activity. Also, treatment during post-inoculation for 46 hr (+2 hr) suppressed HCV infectivity in culture supernatants, though to a lesser extent compared to pre-treatment of virus for 2 hr (−2 hr virus). The result suggested that this treatment inhibited either HCV replication in the cell or HCV infectivity outside the cells (in culture supernatants). On the other hand, treatment during post-inoculation for 46 hr (+2 hr) did not significantly inhibit HCV NS3 protein accumulation while pre-treatment of virus for 2 hr (−2 hr virus) completely inhibited it at 1 and 2 days post-infection (Figure 2B). We further examined HCV RNA replication and virus infectivity inside the cells. The result revealed that the post-inoculation treatment (+2 hr) did not significantly inhibit HCV RNA replication in the cells (Figure 2C). Moreover, the venom treatment (+2 hr) did not reduce HCV infectivity inside the cells (Figure 2D). Taken together, these results suggest that the *S. maurus palmatus* venom acts directly on HCV particles in culture medium to impair the viral infectivity and that it does not exert its antiviral effect inside the cells.

### Effects of neutralization of the proteinase activity by heating and/or a metalloproteinase inhibitor

**Figure 3:**
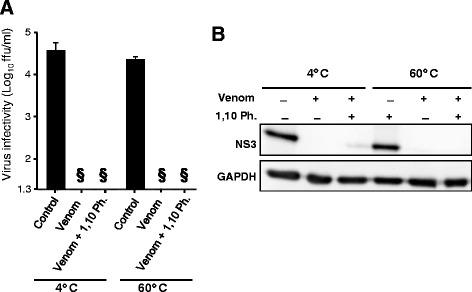
**Effects of neutralization of the proteinase activities of the virocidal effects of** ***S. maurus palmatus*** **venom against HCV.** *S. maurus palmatus* venom (30 μg/ml) was treated with a metalloproteinase inhibitor (1, 10-phenan-throline; 5 mM) at 4°C or 60°C for 20 min. The treated venom was mixed with HCV for 2 hr at 37°C and the mixture was inoculated to Huh7.5 cells for 2 hr at 37°C. The cells were cultivated in the absence of the venom for one day. The culture supernatants were titrated for virus infectivity **(A)** and the cells were subjected to immunoblot analysis using monoclonal antibody against the HCV NS3 protein **(B)**. GAPDH served as an internal control to verify equal amounts of sample loading. Data represent means ± SEM of the data obtained from two independent experiments. 1,10 Ph., 1,10-phenanthroline; §, below the detection limit.

In order to investigate whether anti-HCV activity of *S. maurus palmatus* venom involves an enzymatic activity, we treated the venom (30 μg/ml) with heating at 60°C for 20 min or a metalloproteinase inhibitor, 1, 10-phenan-throline (5 mM) to inactivate them, as reported by other investigators [31-34]. The treated venom was added to HCV and incubated for 2 hr at 37°C. Then, the virus/venom mixture was inoculated to Huh7.5 cells and virus replication was analyzed. The results obtained revealed that the treated venom, either treated with heating at 60°C or the metalloproteinase inhibitor, or both at the same time, still markedly suppressed production of HCV infectious particles in the culture to the same extent compared to the untreated control (Figure 3A). Consistent with this observation, accumulation of intracellular HCV NS3 protein was also inhibited (Figure 3B).

### Specificity of antiviral activity of *S. maurus palmatus* venom

**Figure 4:**
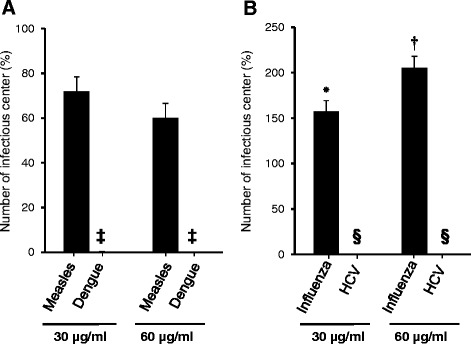
**Antiviral activity of** ***S. maurus palmatus*** **venom against dengue virus, measles virus and influenza virus. (A)** Dengue virus and measles virus that had been treated with *S. maurus palmatus* venom (30 and 60 μg/ml) for 2 hr or left untreated as a control were inoculated to Vero/SLAM cells and cultivated for 24 hr. The number of virus-infected cells was determined by immunofluorescence and plaque assays and the percentages compared to the untreated control calculated. Data represent means ± SD of the data obtained from triplicate cultures. ‡, ~0.2% of the control. **(B)** Influenza virus and HCV that had been treated with *S. maurus palmatus* venom (30 and 60 μg/ml) for 2 hr or left untreated were inoculated to MDCK and Huh7.5 cells, respectively. The number of virus-infected cells was determined by immunofluorescence analysis and the percentages of the numbers of virus-infected cells compared to the untreated control calculated. Data represent means ± SEM of the data obtained from two independent experiments. *, P <0.05; †, P <0.001, compared with the untreated control. §, <0.01% of the control.

In order to determine whether or not the antiviral activity of *S. maurus palmatus* venom shown above was specific to HCV, we examined its possible effects on different viruses, such as dengue virus type 2 [35,36], measles virus [37] and influenza virus [38]. In this analysis, each virus was pre-treated with the venom (30 and 60 μg/ml) for 2 hr and the remaining virus infectivity was measured by infectious center or plaque assay. The result revealed that the venom exerted only weak inhibition on measles virus but strong inhibition on dengue virus (Figure 4A). Interestingly, the same venom did not inhibit but rather enhanced infectivity of influenza virus (Figure 4B).

## Discussion

Venomous animals including scorpions have evolved a wide variety of peptide toxins for the purpose of predation and defense [39]. Scorpion venoms are a rich source of natural peptides and have been recognized as potential bioactive peptides [24,39,40]. cDNA library sequencing and proteomics profiling analyses have revealed that a single scorpion venom contains more than 100 peptidic components ranging in size from 1 to 9 kDa [41,42]. An increasing number of studies have shown that scorpion venoms and toxins possess antiviral activities *in vitro* and *in vivo* and are considered as a rich source for developing effective antiviral drugs [24,26-28,43,44].

In the present study, we screened crude venoms obtained from five Egyptian scorpion species for their possible anti-HCV activities. We observed that crude venoms of *S. maurus palmatus* and *A. australis* possessed anti-HCV activities, with their IC_50_ values being 6.3 ± 1.6 and 88.3 ± 5.8 μg/ml, respectively (Table 1). We also demonstrated that *S. maurus palmatus* venom acts directly on HCV particles in culture supernatants to inhibit the viral infectivity (Figure 2A), suggesting the inhibition at the entry step, the first step of HCV life cycle. On the other hand, it was unlikely that the venom exerted its antiviral activity inside the cells (Figure 2B-D).

Scorpion venoms contain a wide variety of pharmacologically active peptides and proteins. Some of them possess enzymatic activities, such as metalloproteases, while others are non-enzymatic constituents [18,45,46]. It has been reported that the proteolytic activities of those enzymes were almost completely abolished by treatment with a matrix metalloproteinase inhibitor, 1, 10-phenanthroline [31,32,47]. Also, those enzymatic activities are known to be inactivated by heating at 60°C for 20 min [33,34,48]. We made use of this information in our study and observed that treatment of the *S. maurus palmatus* venom with 1, 10-phenanthroline and/or heating at 60°C for 20 min did not impair its anti-HCV activity (Figure 3). These results suggest that the anti-HCV activity of the *S. maurus palmatus* venom is independent of its proteinase activities.

Concerning the molecular mechanism(s) of the anti-HCV activity, there was a possibility that venom peptides induced disruption in virus envelope (composed of lipid bilayer) through making pores in it. If this was the case, the venom might inhibit other envelope viruses than HCV. To test this possibility, we used three different viruses, such as dengue virus type 2 [35,36], another member of the family *Flaviviridae*, and measles virus [37] and influenza virus [38] that belong to the family *Paramyxoviridae* and *Orthomyxoviridae*, respectively. The results obtained demonstrated that *S. maurus palmatus* venom exerted strong inhibition on dengue virus and only weak inhibition on measles virus (Figure 4A). On the other hand, the same venom did not inhibit but rather enhanced influenza virus infectivity (Figure 4B). These results exclude the possibility that *S. maurus palmatus* venom inhibits all the envelope viruses, suggesting that the venom preferentially inhibits HCV and dengue virus, both of which belong to the family *Flaviviridae*, but not other viruses, e.g., influenza virus.

*S. maurus palmatus* venom has been reported to contain about 65 compounds, whose molecular masses vary from 413 to 14,009 Da, with a majority ranging between 3 and 5 kDa. A variety of putative bioactive molecules have been identified, such as neurotoxins (NaScTxs and KScTxs), calcines, La1-like peptides, insecticidal toxins and other AMPs [40]. Some of the scorpion venom peptides showed antiviral activities against certain viruses, such as measles virus, SARS coronavirus, H5N1 influenza virus [27], hepatitis B virus [49], herpes simplex virus 1 [28] and human immunodeficiency virus [50]. As for anti-HCV peptides from scorpion venoms, Yan et al. [26] reported that Hp1090 screened from the venomous gland cDNA library of the scorpion *Heterometrus petersii* inhibited HCV infection by targeting the viral membrane, disrupting its structural integrity. Also, Hong et al. [43] identified another anti-HCV peptide Ctry2459 from the venom peptide library of the scorpion *Chaerilus tryznai.* These peptides exerted a virocidal effect on HCV and some other viruses. Consistent with those results, we observed that *S. maurus palmatus* venom inhibited infectivity of HCV particles, suggesting direct virocidal activity of the venom. Interestingly, the antiviral activity of *S. maurus palmatus* venom is likely to be preferentially directed to HCV and dengue virus, both of which are members of the family *Flaviviridae*. Further studies using bioactivity-guided fractionation and purification analyses are needed to identify an active compound(s) responsible for this antiviral activity.

## Conclusions

We screened crude venoms obtained from five Egyptian scorpion species for anti-HCV activities and demonstrated that *S. maurus palmatus* venom inhibits HCV infectivity through direct virocidal activity. In addition, this antiviral activity appeared to be independent of proteinase activities of the venom and is directed preferentially against HCV, but not equally against all the enveloped viruses. To our knowledge, this is the first report describing antiviral activities of Egyptian scorpion venoms against HCV, and has opened a new approach towards discovering antiviral compounds derived from scorpion venoms.

## Methods

### Scorpions and venom preparation

Adult scorpions were collected from different places in Egypt. They were fed with cockroaches and received water. The venoms were obtained from scorpions using electrical stimulation (12 to 20 V) or by surgically separating venom glands from smaller scorpions, with the milked venoms being squeezed out into an Eppendorf tube using fine forceps, and solubilized in distilled water. The crude venoms thus obtained were centrifuged at 14,000 rpm for 10 min at 4°C. The supernatants were pooled, freeze-dried and stored at −20°C. The lyophilized samples were dissolved in distilled water, centrifuged at 15,000 rpm for 15 min at 4°C and the supernatants were stored at −20°C until being used. Protein concentrations of the samples were determined using BCA Protein Assay Kit (Pierice Biotechnology, USA).

### Cell culture and viruses

Huh7.5 cells and the plasmid pFL-J6/JFH1 to produce the J6/JFH1 strain of HCV genotype 2a [51] were kindly provided by Dr. C. M. Rice, The Rockefeller University, New York, NY, USA. Huh7.5 cells were cultivated in Dulbecco’s modified Eagle’s medium (Wako, Osaka, Japan) supplemented with fetal bovine serum (Biowest, Nuaille, France), non-essential amino acids (Invitrogen, Carlsbad, CA, USA), penicillin (100 IU/ml) and streptomycin (100 μg/ml) (Invitrogen). Cells were grown at 37°C in a 5% CO_2_ incubator. The J6/JFH1-P47 strain [51] of HCV genotype 2a propagated in Huh7.5 cells was used in this study.

Dengue virus type 2 (Trinidad 1751 strain) [35,36], propagated in BHK-21 cells, was inoculated to Vero/SLAM cells [52]. After virus adsorption for 1 hr, the virus-infected cells were cultivated in DMEM supplemented with 10% fetal bovine serum at 37°C in 5% CO_2_.

Measles virus (K52 strain) [37], propagated in B95a cells, was inoculated to Vero/SLAM cells. After virus adsorption for 1 hr, the virus-infected cells were cultivated as described above.

Influenza A virus A/Udorn/307/72 (H3N2) was inoculated to Madin-Darby canine kidney (MDCK) cells for 60 min on ice and the virus-infected cells were cultivated in minimum essential medium (MEM; Life Technologies, Tokyo, Japan) containing 2.5 μg/ml TPCK-trypsin (Worthington Biochemical Corp., Lakewood, NJ, USA) and 100 units/ml each of penicillin and streptomycin (GIBCO, Gland Island, NY, USA) at 37°C in 5% CO_2._

### Analysis of antiviral activities of crude venoms

Huh7.5 cells were seeded in 24-well plates (1.6 × 10^5^ cells/well). A fixed amount of HCV was mixed with serial dilutions of crude venoms (0.1 to 100 μg/ml) and inoculated to the cells. After 2 hr, the cells were washed with medium to remove the residual virus and further incubated in medium containing the same concentrations of the crude venoms as those used during virus inoculation. Culture supernatants were obtained at 1 and 2 days post-infection (dpi) and titrated for virus infectivity. Virus and cells treated with medium served as controls. Percent inhibition of virus infectivity by the samples was calculated by comparing with the controls and 50% inhibitory concentrations (IC_50_) were determined.

### Virus titration

HCV infectivity was determined as described previously [53]. In brief, virus samples were diluted serially 10-fold in complete medium and inoculated onto Huh7.5 cells seeded on glass coverslips in a 24-well plate. After virus adsorption for 2 hr, the cells were washed with medium to remove residual virus and cultured for 24 hr. The virus-infected cells were washed with phosphate-buffered saline (PBS), fixed with 4% paraformaldehyde for 20 min and permeabilized with 0.1% Triton X-100 in PBS for 15 min at room temperature. After being washed three times with PBS, the cells were incubated with HCV-infected patient’s serum for 1 hr, followed by incubation with FITC-conjugated goat anti-human IgG (Medical & Biological Laboratories Co., Ltd., Nagoya, Japan). The cells were counterstained with Hoechst 33342 (Molecular Probes, Eugene, OR, USA) for 5 min and HCV-infected cells were counted under a BZ-9000 fluorescence microscope (Keyence, Osaka, Japan).

To determine intracellular HCV infectivity, freeze-and-thaw experiments were performed. In brief, virus-infected cells were washed with PBS, harvested by trypsin treatment and centrifuged at 1,250 rpm for 5 min at 20°C. The cell pellets obtained were resuspended in 400 μl of fresh medium and subjected to 3 cycles of freezing and thawing in a thermo block at −80°C and 37°C, respectively. The samples were centrifuged at 3,000 rpm for 10 min at 4°C to remove cell debris, and HCV infectivity was measured by immunofluorescence staining, as described above.

To determine dengue virus infectivity, serially diluted virus was inoculated to Vero/SLAM cells and cultivated for 24 hr. The cells were subjected to indirect immunofluorescence analysis using mouse monoclonal antibody against dengue virus followed by Alexa Fluor A488 goat anti-mouse IgG (Life Technologies), as described above, and the number of infected cells counted.

Measles virus was inoculated to Vero/SLAM cells and cultivated for 24 hr. Plaques (virus-induced syncytia) forming on the monolayer cells were counted.

Influenza virus was inoculated to MDCK cells and cultivated for 6 ~ 8 hr. The cells were fixed with 4% paraformaldehyde for 10 min followed by cold methanol (−30°C) for 5 min at room temperature. The fixed cells were incubated for 30 min with rabbit polyclonal antibody raised against purified influenza virus (A/Udorn/307/72 strain) [38]. After two washes, the cells were incubated for 30 min with Alexa Fluor A488 goat anti-rabbit IgG (Life Technologies) and Hoechst 33342 (to counterstain nuclei of all cells). The coverslips were mounted on a slide glass with Vectashield H-1000 reagent (Vector Laboratories, Inc. Burligame, CA) and observed using a fluorescence microscope.

### Time-of-addition experiments

The crude venom was added at a final concentration of 30 μg/ml to the virus or cells at different time points relative to virus inoculation. Five sets of experiments were performed in parallel: (i) Pre-treatment of cells (−2 hr cells): venom was added to the cells for 2 hr at 37°C followed by washing two times with medium before virus infection and incubated with medium without venom for 46 hr. (ii) Pre-treatment of virus (−2 hr virus): the virus was mixed with the venom for 2 hr at 37°C, and then the mixture was inoculated to the cells for 2 hr at 37°C. After the virus/venom mixture was removed by washing two times, the cells were incubated with medium without venom for 46 hr. (iii) Co-treatment of cells and virus during virus inoculation (0 hr): Virus/venom mixture was inoculated to the cells and incubated for 2 hr. After the virus/venom mixture was removed by washing two times, the cells were incubated with medium without venom for 46 hr. (iv) Treatment during post-inoculation (+2 hr): Cells were infected with the virus for 2 hr in the absence of the venom. After the virus was removed by washing two times, the cells were incubated with medium containing the venom for 46 hr. (v) Co-treatment during inoculation and post-inoculation (0 hr & +2 hr): As a positive control, virus/venom mixture was inoculated to the cells and incubated for 2 hr. After the virus/venom mixture was removed by washing two times, the cells were incubated with medium containing the venom for 46 hr. In each set of experiments, the supernatants were collected and titrated for virus infectivity. Also, the cells were subjected to immunoblot analysis for intracellular accumulation of viral and host proteins.

### WST-1 assay for cytotoxicity test

WST-1 assay was performed as described previously with a slight modification [53]. In brief, Huh7.5 cells plated in 96-well plates were treated with serial dilutions of crude venoms or complete medium as a control for 48 hr at 37°C. After this treatment, 10 μl of WST-1 reagent (Roche, Mannheim, Germany) was added to each well and the cells were cultured for 4 hr. The WST-1 reagent is absorbed by the cells and converted to formazan by mitochondrial dehydrogenases. The amount of formazan, which correlates with the number of living cells, was determined by measuring the absorbance of each well using a microplate reader at 450 and 630 nm. Percent cell viability compared to the control was calculated for each dilution of the venoms and 50% cytotoxic concentrations (CC_50_) were determined.

### Immunoblot analysis

Cells were lysed with SDS sample buffer and equal amounts of protein were subjected to SDS–polyacrylamide gel electrophoresis. The separated proteins were transferred onto a polyvinylidene difluoride membrane (Millipore, Bedford, MA, USA). The membrane was blocked by incubation with 5% skim milk and incubated with the respective primary antibodies. The primary antibodies used were mouse monoclonal antibodies against HCV NS3 and GAPDH (Millipore). Horseradish peroxidase-conjugated goat anti-mouse immunoglobulin (Invitrogen) was used to visualize the respective proteins by means of an enhanced chemiluminescence detection system (ECL; GE Healthcare, Buckinghamshire, UK).

### Real-time quantitative RT-PCR

Total RNA was extracted from the cells using a ReliaPrep RNA cell miniprep system (Promega, Madison, WI, USA) according to the manufacturer’s instructions. One μg of total RNA was reverse transcribed using a GoScript Reverse Transcription system (Promega) with random primers and subjected to quantitative real-time PCR analysis using SYBR Premix Ex Taq (TaKaRa, Kyoto, Japan) in a MicroAmp 96-well reaction plate and an ABI PRISM 7500 system (Applied Biosystems, Foster City, CA, USA). The primers used to amplify an NS5A region of the HCV genome were 5′-AGACGTATTGAGGT CCATGC-3′ (sense) and 5′ CCGCAGCGACGGTGCT GATAG-3′ (antisense). As an internal control, human glyceraldehyde-3-phosphate dehydrogenase (GAPDH) gene expression levels were measured using primers 5′ GCCATCAATGACCCCTTCATT-3′ (sense) and 5′ TCTCGCTCCTGGAAGATGG-3′.

### Neutralization of the proteinase activities of scorpion crude venom by heating and a metalloproteinase inhibitor

Crude venom (30 μg/ml) was heated at 60°C for 20 min or treated with a metalloprotease inhibitor (1,10-phenanthroline; 5 mM) [31-34] at 4°C or 60°C for 20 min. The treated venom or untreated control was mixed with HCV for 2 hr at 37°C. The virus/venom mixture was then inoculated to Huh7.5 cells for 2 hr at 37°C. After the virus inoculation, the cells were washed three times and incubated with medium without venom. After 48 hr, culture supernatants were collected and virus infectivity was titrated. The virus-infected cells were subjected to immunoblot analysis to check the level of HCV protein accumulation, as described above.

### Statistical analysis

Data are representative of at least 2 independently repeated experiments and presented as mean ± SEM. The statistical significance was examined using Student’s t-test. *P* value of <0.05 was considered significant.

## Abbreviations

HCV: Hepatitis C virus
SVR: Sustained virological response
AMPs: Antimicrobial peptides
CC_50_: 50% cytotoxic concentration
IC_50_: 50% inhibitory concentration
SI: Selectivity index
1, 10 Ph: 1,10-phenanthroline
